# Extracellular vesicles derived from mesenchymal stromal cells mediate endogenous cell growth and migration via the CXCL5 and CXCL6/CXCR2 axes and repair menisci

**DOI:** 10.1186/s13287-021-02481-9

**Published:** 2021-07-22

**Authors:** Kazumasa Kawata, Hideyuki Koga, Kunikazu Tsuji, Kazumasa Miyatake, Yusuke Nakagawa, Takanori Yokota, Ichiro Sekiya, Hiroki Katagiri

**Affiliations:** 1 Department of Joint Surgery and Sports Medicine, Graduate School of Medical and Dental Sciences, Tokyo Medical and Dental University (TMDU), 1-5-45 Yushima, Bunkyo-ku, Tokyo, 113-8519 Japan; 2 Department of Neurology and Neurological Science, Graduate School of Medical and Dental Sciences and Center for Brain Integration Research, Tokyo Medical and Dental University (TMDU), 1-5-45 Yushima, Bunkyo-Ku, Tokyo, 113-8519 Japan; 3 Center for Stem Cell and Regenerative Medicine, Tokyo Medical and Dental University (TMDU), 1-5-45 Yushima, Bunkyo-ku, Tokyo, 113-8519 Japan; 4 Department of Orthopedics, Dokkyo Medical University Saitama Medical Center, 2-1-50 Minamikoshigaya, Koshigaya, Saitama, 343-8555 Japan

**Keywords:** Extracellular vesicles, Meniscus regeneration, Mesenchymal stromal cell, RNA sequencing, CXCR2

## Abstract

**Background:**

Mesenchymal stromal cell-derived extracellular vesicles (MSC-EVs) are promising candidates for tissue regeneration therapy. However, the therapeutic efficacy of MSC-EVs for meniscus regeneration is uncertain, and the mechanisms underlying MSC-EV-mediated tissue regeneration have not been fully elucidated. The aims of this study were to evaluate the therapeutic efficacy of intra-articular MSC-EV injection in a meniscus defect model and elucidate the mechanism underlying MSC-EV-mediated tissue regeneration via combined bioinformatic analyses.

**Methods:**

MSC-EVs were isolated from human synovial MSC culture supernatants via ultrafiltration. To evaluate the meniscus regeneration ability, MSC-EVs were injected intra-articularly in the mouse meniscus defect model immediately after meniscus resection and weekly thereafter. After 1 and 3 weeks, their knees were excised for histological and immunohistochemical evaluations. To investigate the mechanisms through which MSC-EVs accelerate meniscus regeneration, cell growth, migration, and chondrogenesis assays were performed using treated and untreated chondrocytes and synovial MSCs with or without MSC-EVs. RNA sequencing assessed the gene expression profile of chondrocytes stimulated by MSC-EVs. Antagonists of the human chemokine CXCR2 receptor (SB265610) were used to determine the role of CXCR2 on chondrocyte cell growth and migration induced by MSC-EVs.

**Results:**

In the meniscus defect model, MSC-EV injection accelerated meniscus regeneration and normalized the morphology and composition of the repaired tissue. MSC-EVs stimulated chondrocyte and synovial MSC cell growth and migration. RNA sequencing revealed that MSC-EVs induced 168 differentially expressed genes in the chondrocytes and significantly upregulated CXCL5 and CXCL6 in chondrocytes and synovial MSCs. Suppression of CXCL5 and CXCL6 and antagonism of the CXCR2 receptor binding CXCL5 and CXCL6 negated the influence of MSC-EVs on chondrocyte cell growth and migration.

**Conclusions:**

Intra-articular MSC-EV administration repaired meniscus defects and augmented chondrocyte and synovial MSC cell growth and migration. Comprehensive transcriptome/RNA sequencing data confirmed that MSC-EVs upregulated CXCL5 and CXCL6 in chondrocytes and mediated the cell growth and migration of these cells via the CXCR2 axis.

**Supplementary Information:**

The online version contains supplementary material available at 10.1186/s13287-021-02481-9.

## Background

Meniscus tears and defects alter the biomechanical function and accelerate the progression of osteoarthritis (OA) [1–4]. The intact meniscus plays an important role in load bearing. Hence, meniscus tears and defects decrease transmission function and increase tibiofemoral contact pressure by ≤ 2-fold [1, 5, 6]. Moreover, meniscus tears and defects increase the risk of OA by 2–8-fold compared to that in the general population without meniscus tears [7, 8]. Knee replacement using an artificial joint is a treatment option for patients with end-stage knee OA, and more than 790,000 knee replacements are performed each year in the USA. Consequently, the orthopedic community has emphasized the importance of “saving the meniscus” [9, 10]. However, this tissue has limited intrinsic capacity for regeneration because of the low cellularity of endogenous recruited cells. Additionally, no disease-modifying treatment can regenerate meniscus defects and tears, highlighting the need for novel technologies.

Mesenchymal stromal cells (MSCs) have been shown to be promising candidates for meniscus regeneration therapy in an animal model [11, 12]. The regeneration capacity of MSCs has been attributed to secreted trophic factors, such as extracellular vesicles (EVs) [13]. EVs are nano-sized membrane vesicles comprising bioactive lipids, nucleic acids (mRNAs and microRNAs), and proteins, which play vital roles in cell-cell communication [14]. EVs lack immunogenicity; thus, they demonstrate potential as a cell-free drug for tissue regeneration in an animal model [15]. Furthermore, they have displayed therapeutic efficacy against ischemic heart disease [16] and kidney disease [17] and promoted wound healing [18] and cartilage regeneration [19]. However, the therapeutic efficacy of EVs in meniscus regeneration is uncertain.

Studies have investigated the mechanism of EV-induced tissue regeneration. Possible modes of action include enhanced cell viability and growth [20], reduced apoptosis [21], and modulation of inflammatory responses [22]. However, these mechanisms of action have not been fully elucidated [23] and therefore need to be clarified in order to optimize the clinical use of EVs.

Here, we evaluated the therapeutic efficacy of an intra-articular MSC-EV injection in a mouse model of meniscus defect. We analyzed the mechanism of EV-mediated tissue regeneration using combined bioinformatics analyses. The mouse meniscus defect model has been used to generate proof-of-concept data for meniscus regeneration [11]. Chondrocytes are the main components of the meniscus [24], and endogenous synovial MSCs infiltrate regenerated meniscus tissue [25]. Therefore, we performed in vivo and in vitro experiments using chondrocytes and synovial MSCs. In addition, combined bioinformatics analyses were performed based on the gene expression data from chondrocytes and synovial MSCs.

## Methods

### Preparation of synovial MSCs and chondrocytes

All human procedures were approved by the Local Ethical Committee for Human Research (Tokyo Medical and Dental University, Tokyo, Japan; Approval No.2121). Synovial MSCs were isolated and cultured from the synovium, as previously described [26]. Briefly, the synovia were harvested from patients who provided written, informed consent to participate in this research prior to undergoing anterior cruciate ligament reconstruction. Participants comprised four males and one female. The mean patient age was 22.0 ± 4.56 years. Synovial MSCs were isolated from the synovium by immersing in collagenase and dispase for 3 h followed by filtration through a 70 μm mesh (Becton Dickinson, Franklin Lakes, NJ, USA). The digested cells were incubated in a complete culture medium (alpha minimal essential medium [MEM] supplemented with 10% [v/v], fetal bovine serum [FBS], and 1% [w/v] penicillin-streptomycin-amphotericin B [Invitrogen; Thermo Fisher Scientific, Waltham, MA, USA]) under a humidified 5% CO_2_ atmosphere at 37 °C. Synovial MSCs were used at passages 3–6 in all experiments. Chondrocytes were isolated from cartilage tissues as previously described [27]. Briefly, the cartilage tissue was harvested from patients who had undergone total knee arthroplasty. The cartilage tissues were digested in 3 mg/mL collagenase for 6 h. The nucleated cells were filtered, plated at 10^4^ cells per 60 cm^2^ dish, and cultured as passage 0 for 14 days. The cells were cultured in Dulbecco’s modified Eagle’s medium (DMEM)-F12 supplemented with 5% (v/v) FBS, 5 mg/mL ITS + Premix (Becton Dickinson), and 1% (w/v) penicillin-streptomycin-amphotericin B (Invitrogen; Thermo Fisher Scientific) under a humidified 5% CO_2_ atmosphere at 3 °C. Chondrocytes were used at passages 3–5 in all experiments.

### Differentiation assay of synovial MSCs

Calcification induction culture was conducted as previously described [28]. Briefly, 100 cells were cultured for 14 days in a complete culture medium on a 60-cm^2^ dish. Consequently, these adherent cells were cultured in an osteogenic induction medium (50 μg/mL ascorbic acid 2-phosphate [Sigma-Aldrich, St. Louis, MO, USA], 10 nM dexamethasone [Sigma-Aldrich], and 10 mM β-glycerophosphate [Sigma-Aldrich], in a complete culture medium). The osteogenic induction medium was changed every 3–4 days. After 21 days, alizarin red staining (EMD Millipore, Billerica, MA, USA) confirmed the differentiation of these cells into osteoblasts. Adipogenic induction culture was performed as previously described [28]. Adherent cells were cultured in the adipogenic induction medium (100 nM dexamethasone [Sigma-Aldrich], 0.5 mM isobutylmethylxanthine [Sigma-Aldrich], and 100 mM indomethacin [Wako Pure Chemical Industries Ltd., Osaka, Japan] in a complete culture medium). The adipogenic induction medium was changed every 3–4 days. After 21 days, the differentiation of these cells into adipocytes was assessed using oil red-O staining (Muto Pure Chemicals, Tokyo, Japan). Chondrogenic differentiation by pellet culture was conducted as previously described [26]. Briefly, 1.5 × 10^6^ synovial MSCs were cultured in a 15-mL polypropylene tube (Becton Dickinson) and centrifuged at 1500 rpm for 10 min to form pellets. The pellets were then cultured in a 400-μL chondrogenesis medium for 21 days. The pellets were fixed, embedded in paraffin, cut into 5 μm sections, and stained with Toluidine Blue for histological evaluation.

### Flow cytometric analysis of synovial MSCs

Cell surface molecules were analyzed using a flow cytometer (FACSVerse TM, BD Biosciences, Tokyo, Japan). Briefly, cultured cells (1–5 × 10^5^ cells/mL) were washed twice with FACS staining buffer and counted. Cells (100,000) were incubated with antibodies targeting the molecules or isotype of interest. Detailed information on each antibody is provided in Supplementary Table 1. Isotypes were used to establish staining specificity. All analyses were performed using BD FACSVerse TM and BD FACSSuite TM Software (BD Biosciences, Tokyo, Japan).

### MSC-EV isolation and identification

Synovial MSCs were seeded at 1.5 × 10^6^ cells per 15-cm^2^ plate and incubated in a complete culture medium for 24 h. The cells were then washed twice with phosphate-buffered saline (PBS) and cultured with alpha MEM containing 5% exosome-depleted FBS (SBI, Palo Alto, CA, USA) or serum-free alpha MEM for 48 h. The cell suspension was centrifuged at 2000×*g* and 4 °C for 10 min to remove detached cells. The supernatant was collected and passed through a 0.22-μm filter (EMD Millipore, Billerica, MA, USA) to remove cellular debris. The filtered supernatant was then transferred to the upper compartment of a 10-kDa Amicon Ultra Filter Unit (EMD Millipore, Billerica, MA, USA) and centrifuged at 4000×*g* and 4 °C for 30 min. The liquid was washed with PBS and subjected to ultrafiltration. The MSC-EVs were collected from the upper compartment and stored in a PROKEEP low-protein binding tube (WATSON, Tokyo, Japan) at − 80 °C. Nanoparticle tracking analysis (NTA) was performed on the EVs using NanoSight LM10 and NanoSight NTA v3.0 (Malvern Panalytical, Malvern, UK). Western blotting was used to identify the EV-associated markers CD9 and CD63. MSC-EVs were negatively stained with 2% (w/v) uranyl acetate, and their morphology was observed under a transmission electron microscope (TEM; JEM-1400 Flash; JEOL Ltd., Tokyo, Japan).

### Cell growth assay

The cell growth assay was conducted as previously described [29]. Chondrocytes and synovial MSCs were seeded in a 96-well plate at a density of 1 × 10^5^ cells/well. The cells were treated with either 2 × 10^9^ particles/mL MSC-EVs or PBS vehicle control. Cell growth was measured on days 0, 3, 7, 10, and 13, with a Cell Counting Kit-8 (CCK-8; Dojindo, Kumamoto, Japan) according to the manufacturer’s instructions. Ten microliters of CCK-8 reagent was added to each well, and the plate was incubated at 37 °C for 1.5 h. Absorbances were measured at 450 nm in an Infinite® F50 Absorbance Microplate Reader (Tecan Trading A, Männedorf, Switzerland). Chondrocytes and synovial MSCs were cultured in six-well plates using the above methods. The cells were fixed in 4% (v/v) paraformaldehyde (PFA) and stained with 0.5% (w/v) crystal violet (Wako Pure Chemical Industries Ltd.).

### Cell migration assay

The effects of MSC-EVs on the migration of chondrocytes and synovial MSCs were evaluated by a Transwell® migration assay. The upper chamber contained 5 × 10^4^ cells per 100 μL serum-free medium. Each lower chamber contained 500 μL complete culture medium with or without 4 × 10^9^ particles/mL MSC-EVs. Chondrocytes were incubated for 24 h, and synovial MSCs were incubated for 12 h at 37 °C. After incubation, the upper chamber was fixed with 4% (v/v) PFA for 5 min, stained with 0.5% (w/v) crystal violet for 10 min, and washed three times with PBS. The upper surface of the Transwell membrane was wiped with a cotton swab to remove the cells. The cells were counted in five randomly selected fields at × 100 (Olympus BX 53; Olympus, Tokyo, Japan).

### MSC chondrogenesis assay

The effects of the MSC-EVs on synovial MSC chondrogenesis were evaluated by pellet culture, as previously described [26]. Briefly, 1.5 × 10^6^ synovial MSCs were cultured in a 15-mL polypropylene tube (Becton Dickinson) and centrifuged at 1500 rpm for 10 min to form pellets. The pellets were then cultured with either 2 × 10^9^ particles/mL MSC-EVs or PBS in 400 μL chondrogenesis medium (high-glucose Dulbecco’s modified Eagle’s medium (Invitrogen; Thermo Fisher Scientific) supplemented with 1000 ng/mL BMP-7 (Stryker Biotech, Kalamazoo, MI, USA), 10 ng/mL TGF-β3 (R&D Systems, Minneapolis, MN, USA), 100 nmol/L dexamethasone, 50 ng/mL ascorbate-2-phosphate, 40 mg/mL proline, 100 g/mL pyruvate (Sigma-Aldrich), and 50 mg/mL ITS + Premix [Becton Dickinson]) for 21 days. The pellets were fixed, embedded in paraffin, cut into 5-μm sections, and stained with Toluidine Blue for histological evaluation.

### Mouse meniscal defect model

The animal study protocol was approved and conducted in accordance with the guidelines of the Institutional Animal Care and Use Committee of Tokyo Medical and Dental University (Approval No. A2020-132A). Male C57BL/6J mice were purchased from Oriental Yeast Co. Ltd. (Tokyo, Japan), housed in an environmentally controlled animal facility under a 12-h light/12-h dark cycle, and provided with food and water ad libitum. Twenty-four male mice aged 8 weeks were used in the study. Defects were made on the anterior medial menisci, as previously described [30]. Briefly, a straight incision was made on the anterior side of the knee under isoflurane inhalation anesthesia. The joint capsule was cut, and the anterior medial meniscus horn was exposed. The medial meniscus was cut vertically at the medial collateral ligament level and excised under a stereomicroscope (Zeiss Stemi 2000C; Carl Zeiss AG, Oberkochen, Germany). To evaluate MSC-EV regeneration, based on the EV concentration reported previously [29], 10^8^ MSC-EV particles per 10 μL PBS were injected intra-articularly into the left knee (n = 6). Control treatment consisted of 10 μL PBS, which was injected into the contralateral right knees (n = 6). Intra-articular injections were performed immediately after meniscus resection and weekly thereafter. After 1 and 3 weeks, the mice were euthanized by CO_2_ inhalation, and their knees were excised for histological and immunohistochemical evaluations.

### Histological evaluation

Knee samples were fixed in 4% (v/v) PFA for 7 days, decalcified in 20% (w/v) ethylenediaminetetraacetic acid (EDTA) for 10 days, dehydrated, and embedded in paraffin. The samples were cut in the sagittal plane to 5 μm thickness and stained with safranin-O/fast green and hematoxylin and eosin (HE) to evaluate meniscus regeneration with a modified Pauli’s scoring system (Supplemental Table 2) [30]. Safranin-O-positive areas in the estimated post-regeneration menisci were measured using ImageJ v1.52p (National Institutes of Health, Bethesda, MD, USA). The Pauli scores were evaluated by two independent observers blinded to the treatments.

### Immunohistochemical evaluation

Tissue sections were deparaffinized in xylene (Wako Pure Chemical Industries Ltd.), rehydrated in graded alcohol, and saturated with PBS. For type II collagen staining and antigen retrieval, the samples were pretreated with 0.4 mg/mL proteinase K (Agilent Technologies, Santa Clara, CA, USA) in 50 mM Tris-HCl buffer (Wako Pure Chemical Industries Ltd.) for 5 min at room temperature. For proliferative cell nuclear antigen (PCNA) staining, the samples were pretreated with Target Retrieval Solution 10X (× 10 dilution; Agilent Technologies) for 30 min. Endogenous peroxidase was quenched by incubating in 0.3% (v/v) hydrogen peroxide in methanol for 30 min. Tissues were blocked with 5% (v/v) goat or horse serum plus 1% (v/v) BSA in PBS. The slices were incubated with primary antibodies at 4 °C overnight. The primary antibodies comprised mouse anti-type I collagen (1:200 dilution; ab34712; Abcam, Cambridge, UK), mouse anti-type II collagen (1:1000 dilution; ab34712; Abcam), and anti-PCNA (1:1000 dilution; ab53048; Abcam). The tissue sections were rinsed with 0.1% (v/v) PBS-Triton X-100 (MP Biomedicals, Santa Ana, CA, USA) and incubated in 1:200 secondary antibodies. The secondary antibodies included biotinylated goat anti-rabbit IgG for types I and II collagen (Vector Laboratories, Burlingame, CA, USA) and biotinylated horse anti-mouse IgG for anti-PCNA (Vector Laboratories). The tissue sections were rinsed with PBS, and signals were visualized using Vectastain ABC reagent (Vector Laboratories) followed by 3,3′-diaminobenzidine (DAB) staining (Vector Laboratories). The tissue sections were counterstained with hematoxylin (Muto Pure Chemicals). PCNA-positive/negative stained cells in the estimated post-regeneration meniscus were enumerated at × 400 magnification (Olympus BX 53). The cells were counted in three separate areas per sample by two independent observers blinded to the treatments.

### RNA sequencing

Chondrocytes and synovial MSCs were seeded in six-well plates at 1.2 × 10^5^/well, incubated for 48 h, and cultured with 2 × 10^9^ particles/mL MSC-EVs or PBS for 24 h. The cells were subjected to RNA sequencing on a BGISEQ-500 by BGI Co. Ltd. (Beijing, China). Differentially expressed genes (DEGs) were identified as those with Q (adjusted P) ≤ 0.05 according to a previously described method [31]. DEGs were visualized with a volcano diagram plotted using a log2 scale and Q. DEGs were analyzed using the Kyoto Encyclopedia of Genes and Genomes (KEGG) pathway database (http://www.genome.jp/kegg/). Data are shown in Supplementary Table 3.

### Real-time quantitative polymerase chain reaction (qRT-PCR)

Chondrocytes were prepared in the same way as described for RNA sequencing. Total RNA was extracted from cells using a High Pure RNA isolation kit (Roche Diagnostics, Mannheim, Germany), according to the manufacturer’s instructions. The cDNA was synthesized with a Transcriptor First Strand cDNA synthesis kit (Roche Diagnostics). RT-qPCR was performed in a LightCycler 480 Probe Master kit (Roche Diagnostics). Relative mRNA quantities were normalized to those of β-actin, as previously described [32]. The PCR primer sequences are listed in Supplementary Table 4.

### Western blotting

Western blotting was performed as previously described [33]. Briefly, chondrocytes were starved in 0.5% (v/v) FBS medium with antibiotics for 24 h to inhibit phosphorylation, and then treated with 2 × 10^9^ particles/mL MSC-EVs or PBS for ≤ 20 min. The total cell lysate was prepared with cell lysis buffer (#9803S; Cell Signaling Technology, Danvers, MA, USA). Antibodies against phospho-p44/42 (#4370), p44/42 (#9102), phospho-Akt (#9271), and Akt (#9272) were purchased from Cell Signaling Technology.

### Inhibition of CXCR 2, AKT, and ERK pathways

A human chemokine CXCR2 receptor antagonist (SB265610 [#1559]; Axon Medchem, Groningen, The Netherlands), PI3K signaling inhibitor (LY290042 [#129-04861]; Wako Pure Chemical Industries Ltd.), and Erk1/Erk2 signaling inhibitor (PD98059 [#9900]; Cell Signaling Technology) were used to determine the involvement of the CXCR2, AKT, and ERK pathways on the chondrocytes treated with MSC-EVs. Chondrocytes were pre-treated with 20 μM SB265610, 7 μM LY290042, 20 μM PD98059, or dimethyl sulfoxide (DMSO) vehicle for 1 h and subjected to qRT-PCR, western blotting, and cell growth and migration assays, as previously described.

### Statistical analysis

Data were analyzed using GraphPad Prism v. 8 (GraphPad Software, Inc., La Jolla, CA, USA). A Mann-Whitney *U* test was used to compare pairs of treatment means. Statistical significance was set to *P* < 0.05. Data are presented as means ± standard deviation (SD).

## Results

### Characterization of human synovial MSCs and MSC-EVs

**Fig. 1:**
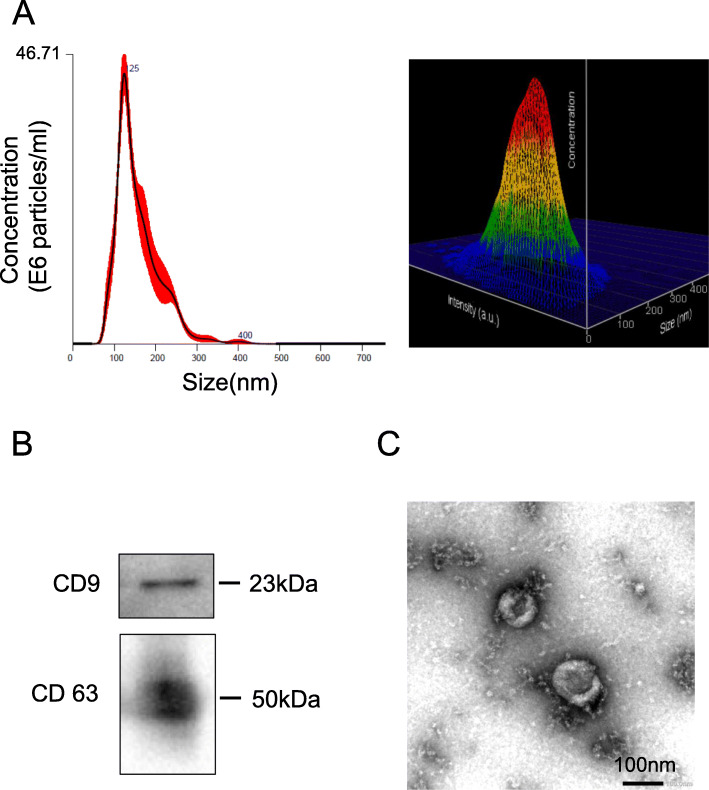
Characterization of MSC-EVs. **A** Size distribution of EVs measured in triplicate by NTA. The red area indicates the SD. The 3D plot indicates the EV size, particle number, and intensity measured by NTA. X-axis: number of particles. Y-axis: intensity. Z-axis: number of particles. **B** Western blotting of EV-associated markers including CD9 and CD63. **C** MSC-EV morphology was observed by TEM at × 50,000

Flow cytometric analysis confirmed that synovial MSCs were positive for CD44, CD73, CD90, and CD105 and negative for CD31 and CD45. The synovial MSCs differentiated into chondrocytes, adipocytes, and calcified (Supplementary Figure 1). Nanoparticle tracking analysis (NTA) showed that the average MSC-EV diameter was ~ 156.7 ± 2.8 nm. The diameter of most MSC-EVs was 124.3 ± 0.4 nm (Fig. 1A). This finding corroborated that of a previous report [34]. Western blotting confirmed that the MSC-EVs expressed the CD9 and CD63 markers (Fig. 1B). TEM showed that the MSC-EVs were nearly spherical and ~ 100 nm in diameter (Fig. 1C).

### Effect of MSC-EVs on meniscus regeneration in a mouse meniscus defect model

**Fig. 2:**
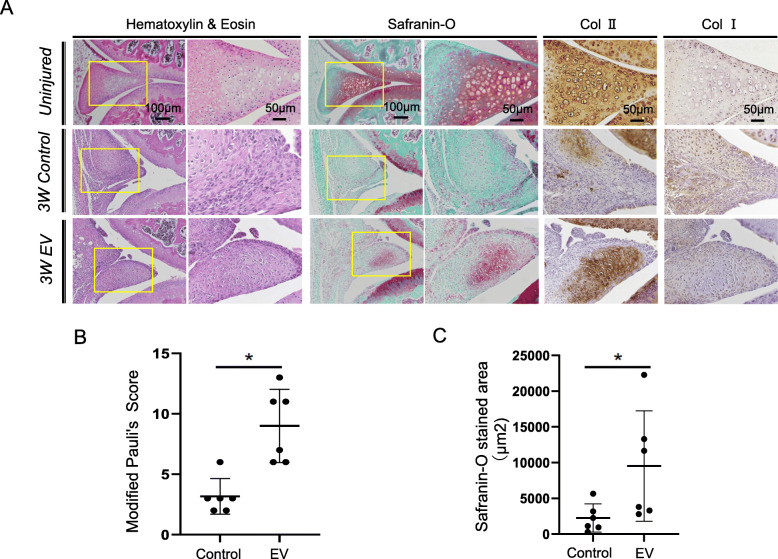
Effect of MSC-EVs on meniscus regeneration. **A** Histological and immunohistochemical (IHC) staining of regenerated menisci with or without MSC EV treatment. Samples were stained with HE, safranin-O/fast green, type II collagen (Col II), and type I collagen (Col I) (n = 6; 3 weeks). **B** Semiquantitative evaluation of meniscus regeneration by modified Pauli’s score. **C** Quantitative evaluation of the safranin-O-positive area by ImageJ. Three sections per knee were evaluated. Data are the means ± SD. *P < 0.05 according to the Mann-Whitney *U* test

In a previous study, mouse meniscus did not spontaneously regenerate 3 weeks after meniscectomy [30]. The surface of the regenerated meniscus was smoother in the EV group compared with that in the control group (Fig. 2A). Safranin-O and type II collagen-positive tissues were observed in the EV group but not in the control group; fibrous type I collagen-positive tissue was observed in the control group. Semiquantitative evaluation by modified Pauli’s score indicated that meniscus regeneration was significantly greater in the EV group than that in the control group (Fig. 2B). Quantitative evaluation by ImageJ showed that the safranin-O-positive area was significantly larger in the EV group compared with that in the control group (Fig. 2C).

### Effects of MSC-EVs on chondrocyte and synovial MSC cell growth, migration, and chondrogenesis

**Fig. 3:**
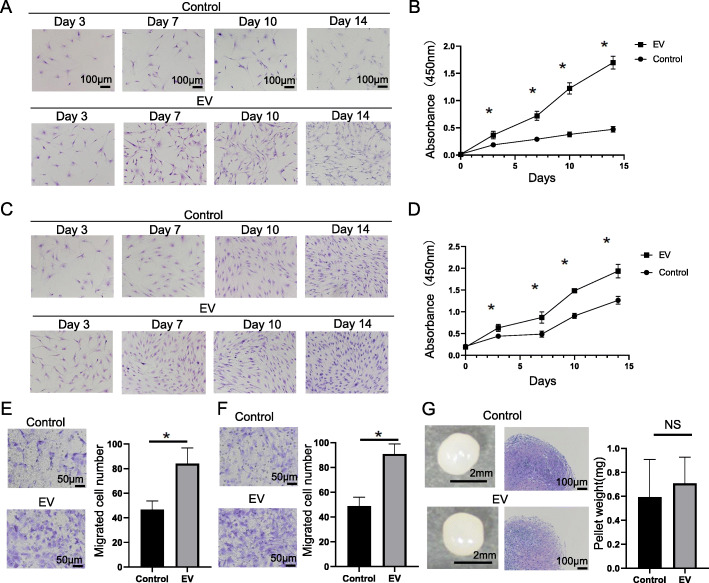
Effects of MSC-EVs on cell growth, migration, and chondrogenesis. **A** Chondrocytes cultured with or without MSC-EVs and stained with crystal violet. **B** CCK-8 cell growth assay in untreated and MSC-EV-treated chondrocytes. Absorbance is proportional to cell number. Measurements were repeated five times, and the average was recorded (n = 6). **C** Synovial MSCs cultured with and without MSC-EVs. **D** CCK-8 assay on synovial MSCs with and without MSC-EV treatment (n = 6). **E** Transwell assay showing the migration of chondrocytes with and without MSC-EV treatment. Chondrocytes migrating to the lower surface were counted in five randomly selected fields (n = 6). **F** Quantitation of migration by synovial MSCs with and without MSC-EV treatment (n = 6). **G** Macroscopic findings and histological Toluidine Blue staining of chondrogenic pellets of synovial MSCs with and without MSC-EV treatment. Pellets were weighed (n = 5; NS, no significant difference; *P* = 0.84). Data are the means ± SD. *P < 0.05 according to the Mann-Whitney *U* test

The main contributors to meniscal regeneration are synovial MSCs and chondrocytes [35]. To investigate the mechanisms through which MSC-EVs accelerate meniscus regeneration, cell growth, migration, and chondrogenesis assays were performed on treated and untreated chondrocytes and synovial MSCs. There were significantly more chondrocytes in the EV group compared with those in the control group (Fig. 3A). The results of the CCK-8 assay indicated that MSC-EV treatment increased absorbance in a time-dependent manner, and that absorbance was proportional to cell number (Fig. 3B). MSC-EVs significantly increased the relative chondrocyte cell growth from day 3 onwards. The absorbance recorded for the EV group was 4 fourfold higher than that of the control group on day 13. Over time, there were more synovial MSCs in the EV group than in the control group (Fig. 3C). The results of the CCK-8 assay indicated that MSC-EVs significantly increased the relative cell growth of synovial MSC from day 3 (Fig. 3D). The absorbance recorded for the EV group was ~ 1.5-fold higher than that of the control on day 13. The results of the Transwell assay indicated that significantly more chondrocytes and synovial MSCs migrated to the lower surface in the EV group compared with those in the control group (Fig. 3E, F). A pellet culture assay was performed to assess the effects of MSC-EVs on synovial MSC chondrogenesis. The results revealed no significant difference between the EV group and the control groups in terms of pellet appearance, size, or weight (Fig. 3G). Histological analysis based on Toluidine Blue staining revealed no obvious difference between treatments in terms of chondrogenic differentiation on the metachromasia, representing acidic mucopolysaccharide deposition.

### Early effect of MSC-EVs on endogenous cell growth and migration in a mouse meniscal defect model

**Fig. 4:**
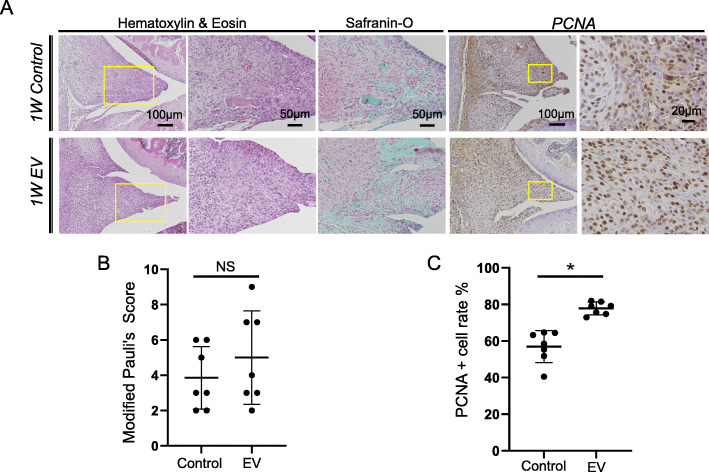
Early effect of MSC-EVs in a mouse meniscal defect model. **A** Histology and IHC staining after 1 week in regenerated meniscus with and without MSC-EV treatment (n = 7). Samples were stained with HE, safranin-O/fast green, and proliferative cell nuclear antigen (PCNA) staining. **B** Semiquantitative evaluation by modified Pauli’s score (NS, no significant difference; *P* = 0.35). Three sections per knee were evaluated, and the average value was calculated. **C** Quantitative evaluation of MSC-EV-induced cell growth. The percentage of PCNA-positive cells in regenerated meniscus was estimated using three sections per knee, and the average was calculated (n = 7). Data are the mean ± SD. *P < 0.05 according to the Mann-Whitney *U* test

The results of the in vitro experiments showed that MSC-EVs enhanced the cell growth and migration of chondrocytes and synovial MSCs. Therefore, we investigated the effects of MSC-EVs on the cell growth and migration of endogenous cells at an early time point in the mouse meniscal defect model. Safranin-O and HE staining revealed no obvious differences between the EV and control groups at week 1 (Fig. 4A). This finding was corroborated by semiquantitative modified Pauli’s score evaluation (Fig. 4B). PCNA staining revealed a higher number of positive proliferative cells in the EV group compared with that in the control group (Fig. 4A). The number of PCNA-positive cells was significantly higher in the EV group than in the control group (Fig. 4C).

### Effects of MSC-EVs on endogenous chondrocyte and synovial MSC gene expression

**Fig. 5:**
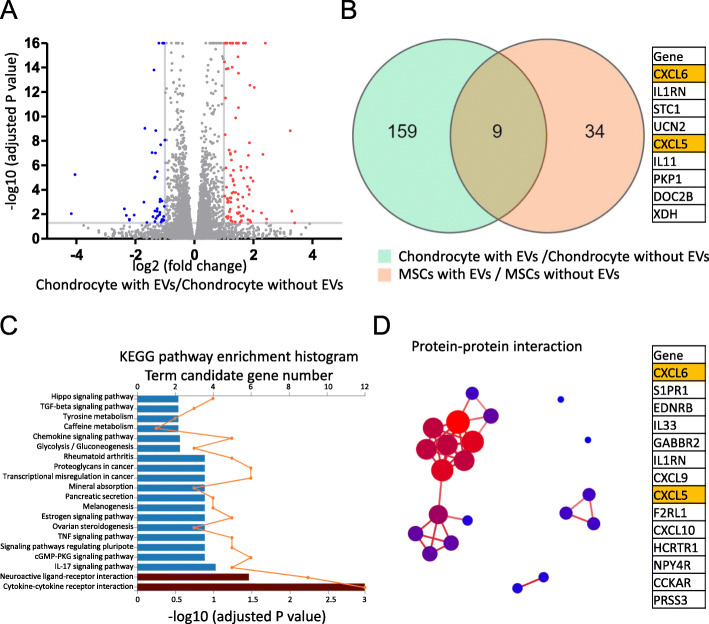
Effects of MSC-EVs on gene expression in chondrocytes and synovial MSCs. **A** Volcano plot showing transcriptome/RNA sequencing data for chondrocytes cultured with and without MSC-EVs for 24 h (n = 4). Red dots represent upregulated DEGs, and blue dots represent downregulated DEGs. **B** Venn diagram of DEGs in chondrocytes and synovial MSCs. Genes common to both are listed in the table. **C** Kyoto Encyclopedia of Genes and Genomes (KEGG) pathway analysis. Bars represent Q, and lines represent candidate gene numbers. **D** Protein-protein interaction on “cytokine-cytokine receptor interaction” and “neuroactive ligand-receptor interaction” gene sets. Symbols for genes composing major clusters are shown in the table

RNA sequencing was used to analyze the early gene expression profile of endogenous chondrocytes stimulated by MSC-EVs. The MSC-EVs modulated 168 DEGs (|fold change| > 2 and adjusted *P* < 0.05) in the chondrocytes; of these, 112 were upregulated and 56 were downregulated (Fig. 5A). To identify robustly expressed genes induced by EVs, the gene expression profiles were compared between treated and untreated synovial MSCs. MSC-EV stimulation induced 43 DEGs in synovial MSCs (Supplementary Figure 1). Nine DEGs were upregulated in both the chondrocytes and synovial MSCs treated with EVs (Fig. 5B).

KEGG pathway enrichment analysis identified the gene sets “cytokine-cytokine receptor interaction” and “neuroactive ligand-receptor interaction” as significantly enriched pathways in the EV-treated chondrocytes (Fig. 5C). KEGG pathway enrichment analysis also identified five gene sets in the EV-treated MSCs (Supplementary Figure 2). A protein-protein interaction database based on the mapping relationship between the STRING11 database output and the NCBI reference transcripts was used to screen for key functional genes in the upregulated gene sets. Fourteen and eight closely related genes were selected through KEGG enrichment and protein-protein interaction analyses in the chondrocytes and synovial MSCs, respectively (Fig. 5D and Supplementary Figure 3). Both methods identified CXCL5 and CXCL6.

### Mechanism of MSC-EV-induced chondrocyte cell growth and migration

**Fig. 6:**
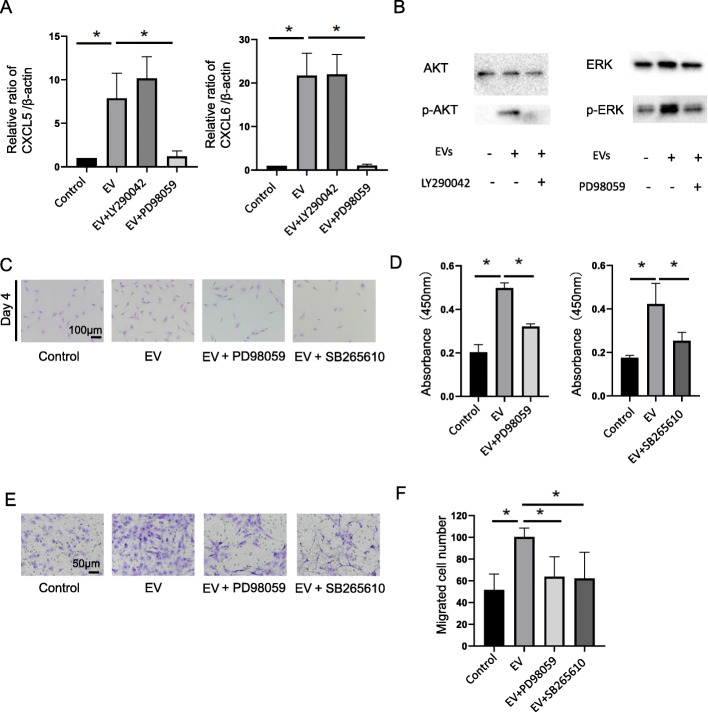
Mechanistic analysis of chondrocyte cell growth and migration induced by MSC-EVs. **A** RT-qPCR analysis of *CXCL5* and *CXCL6* expression in chondrocytes treated with MSC-EVs (n = 6). The data shown represent the fold change relative to the control group. AKT and ERK were inhibited using 7 μM LY290042 and 20 μM PD98059, respectively. **B** Western blotting following AKT and ERK inhibition in response to MSC-EV treatment. **C** Chondrocytes were treated with MSC-EVs and an ERK inhibitor or a CXCR2 antagonist and then stained with crystal violet. **D** CCK-8 cell growth assay of chondrocytes treated with MSC-EVs and an ERK inhibitor or a CXCR2 antagonist. Absorbance corresponds to cell number. Measurements were repeated five times, and the average value was calculated (n = 6). **E** Transwell migration assay of chondrocytes treated with MSC-EVs and an ERK inhibitor or a CXCR2 antagonist. **F** Chondrocytes migrating to the lower surface were counted in five randomly selected fields (n = 6). Data are the mean ± SD. *P < 0.05 according to the Mann-Whitney *U* test

We verified the RNA sequencing results by performing qRT-PCR to analyze CXCL5 and CXCL6 expression in MSC-EV-treated chondrocytes. The expression of CXCL5 and CXCL6 was significantly upregulated in the MSC-EV-treated chondrocytes compared to that in the untreated chondrocytes (Fig. 6A). PI3K/Akt or Erk signaling induces CXCL5 and CXCL6 in multiple cell types [36], and EVs regulate the PI3K/Akt and Erk signaling pathways [37]. To elucidate the signaling pathways through which EVs regulate CXCL5 and CXCL6 expression in chondrocytes, we inhibited MSC-EV-induced AKT and ERK phosphorylation using LY290042 (a PI3K signaling inhibitor) and PD98059 (an Erk1/Erk2 signaling inhibitor) (Fig. 6B). CXCL5 and CXCL6 upregulation by MSC-EVs 24 h post-treatment was suppressed by ERK inhibition, but not by AKT inhibition (Fig. 6A). CXCL5 and CXCL6 suppression following ERK inhibition negated the influence of MSC-EVs on chondrocyte cell growth (Fig. 6C, E). The results of the Transwell migration assay revealed that more chondrocytes treated with MSC-EVs migrated to the lower part compared to those in which CXCL5 and CXCL6 were suppressed by ERK inhibition (Fig. 6F). CXCL5 and CXCL6 activate the chemokine CXCR2 receptor [38]. Chemokine CXCR2 receptor antagonist (SB265610) treatment negated the influence of MSC-EVs on chondrocyte cell growth (Fig. 6D, E). SB265610 abrogated the increase in chondrocyte migration mediated by MSC-EVs (Fig. 6F).

## Discussion

Intra-articular MSC-EV administration has previously been shown to repair cartilage defects [19]. However, studies on the use of MSC-EVs for the treatment of osteoarthritis have focused on cartilage regeneration. To date, no studies have explored MSC-EV therapy for meniscus defects. To the best of our knowledge, the present study is the first to demonstrate the application of MSC-EVs for the management of meniscus damage. Here, we provide empirical evidence for meniscus regeneration by MSC-EVs in a mouse meniscus defect model. There has been a possibility of EVs as a clinical tissue regeneration drug. However, the mechanism through which EVs induce tissue regeneration has not been fully elucidated [39]; this is important to determine in order to optimize the clinical use of MSC-EVs. Two independent bioinformatic analyses were performed based on comprehensive transcriptome/RNA sequencing data, which revealed that MSC-EVs upregulated CXCL5 and CXCL6 in chondrocytes and mediated chondrocyte cell growth and migration via the CXCR2 axis.

The low cellularity of endogenous recruited MSCs and chondrocytes impedes meniscus healing and regeneration [40]. While endogenous MSCs and chondrocytes were found to be successfully recruited for meniscus regeneration in animal models, no disease-modifying treatment is available to regenerate human menisci [41]. EV transplantation may orchestrate cellular processes, such as migration, cell growth, and matrix synthesis, which are associated with tissue regeneration [39, 42]. Previous studies have shown that EVs can increase the cell growth and migration of chondrocytes and regenerate cartilage in the proteomic avascular joint resembling the meniscus [21, 43]. In the current study, EVs promoted the migration and cell growth of synovial MSCs and chondrocytes and meniscus regeneration.

CXCL5 and CXCL6 are neutrophil/granulocyte chemotactic factors and are angiogenic during inflammation. CXCL5 and CXCL6 also induce multipotent progenitor cell growth and migration during tissue homeostasis and healing [44–46], and promote migration, inhibit differentiation, and enhance clonogenicity and self-renewal in MSC [46–48]. In adipocytes and endothelial cells, EVs upregulate CXCL5 and CXCL6 via microRNAs or tetraspanins, which are components of the EVs [49, 50]. Here, RNA sequencing and qRT-PCR analysis confirmed that CXCL5 and CXCL6 were upregulated in chondrocytes and synovial MSCs subjected to MSC-EVs. CXCL5 is induced in fibroblasts and macrophages in response to IL-17 administration. The latter activates the PI3K/Akt and Erk signaling pathways [36]. MicroRNAs and EVs regulate the PI3K/Akt and Erk signaling pathways [37, 51]. In the present study, MSC-EVs induced CXCL5 and CXCL6 in chondrocytes through activation of the Erk signaling pathway.

CXCR2 is a major ELR-CXC chemokine receptor [38], which shares 78% sequence homology with CXCR1; both bind to CXCL6. However, CXCR1 binds to CXCL6 and CXCL8, but not to CXCL5 [52]. CXCR2 interacts with higher-affinity ELR+ chemokines and plays a vital role in cellular chemotaxis [53]. CXCR2 signaling supports cellular migration, survival, and growth [54, 55]. In tissue progenitor cells, CXCR2 signaling contributes to tissue healing, providing insights into cell-based tissue damage therapy [54, 56, 57]. An earlier study showed that CXCR2 signaling in chondrocytes inhibited apoptosis and maintained the chondrocyte phenotype and cartilage homeostasis [44]. Here, we revealed that EVs activated CXCR2 signaling and promoted chondrocyte cell growth and migration.

## Conclusions

The results of this study demonstrate that intra-articular MSC-EV administration repaired meniscus defects and enhanced chondrocyte and synovial MSC cell growth and migration. Comprehensive transcriptome/RNA sequencing data demonstrated that MSC-EVs robustly upregulated CXCL5 and CXCL6 in chondrocytes and mediated the cell growth and migration of these cells via the CXCR2 axis.

## Supplementary Information

**Additional file 1: Fig. S1.** Expression of positive and negative cell-surface markers in mesenchymal stromal cells (MSCs), and their differentiation potential. (A) Representative flow cytometric profiles of colony-forming synovium-derived MSCs stained for CD44, CD73, CD90, and CD105 (positive cell-surface markers), and CD45 and CD31(negative cell-surface markers) (purple: isotype control; red: sample). (B) Chondrogenesis. Histological sections stained with toluidine bule are shown. (C) Adipogenesis. Culture dishes stained with oil red-O are shown. (D) Calcification. Culture dishes stained with alizarin red are shown. **Fig. S2.** Effect of MSC-EVs on meniscus regeneration. Histological and immunohistochemical (IHC) staining of the best and the worst regenerated menisci with or without MSCs EV treatment. Samples were stained with HE, safranin-O/fast green, type II collagen (Col II), and type I collagen (Col I) (n = 6; 3 weeks). **Fig. S3.** Early effect of MSC-EVs in a mouse meniscal defect model. IHC evaluation of proliferative cell nuclear antigen (PCNA) staining after 1 week in the best and the worst regenerated meniscus with and without MSC-EV treatment. **Fig. S4.** Volcano plot presenting the transcriptome/RNA sequencing data for synovial MSCs cultured with or without MSC-EVs for 24 h (n = 4). Gene set enrichment analysis. **Fig. S5.** KEGG pathway analysis of synovial MSCs treated with MSC-EVs. **Fig. S6.** Protein-protein interactions on upregulated gene set in synovial MSCs treated with MSC-EVs.

**Additional file 2: Table S1.** Antibodies used for cytometric analyses.

**Additional file 3: Table S2.** Criteria and scores used for the histological assessment of regenerated menisci (Modified Pauli’s Score, A:3, B:2, C:1, D:0).

**Additional file 4: Table S3.** RNA sequencing data for chondrocytes and synovial MSCs cultured with or without MSC-EVs.

**Additional file 5: Table S4.** Sequences of primers used for RT-qPCR analysis.

## Data Availability

The datasets used and analyzed during the current study are available from the corresponding authors upon reasonable request.

## Abbreviations

MSCs: Mesenchymal stromal cells
EVs: Extracellular vesicles
CXCR2: C-X-C chemokine receptor 2
CXCL: C-X-C motif chemokine ligand
OA: Osteoarthritis
MEM: Minimal essential medium
FBS: Fetal bovine serum
DMEM: Dulbecco’s modified Eagle’s medium
PBS: Phosphate-buffered saline
NTA: Nanoparticle tracking analysis
TEM: Transmission electron microscope
CCK-8: Cell Counting Kit-8
PFA: Paraformaldehyde
BMP-7: Bone morphogenetic protein-7
TGF-β3: Transforming growth factor-β3
EDTA: Ethylenediaminetetraacetic acid
HE: Hematoxylin and eosin
PCNA: Proliferative cell nuclear antigen
BSA: Bovine serum albumin
DAB: Diaminobenzidine
DEGs: Differentially expressed genes
KEGG: Kyoto Encyclopedia of Genes and Genomes
qRT-PCR: Real-time quantitative polymerase chain reaction
cDNA: Complementary DNA
p-AKT: Phospho-AKT
p-ERK: Phospho-extracellular regulated protein kinases
PI3K: Phosphatidylinositol-4,5-bisphosphate 3-kinase
SD: Standard deviation
