# Supplementary material for: Extracellular vesicles derived from mesenchymal stromal cells mediate endogenous cell growth and migration via the CXCL5 and CXCL6/CXCR2 axes and repair menisci
Source: Stem Cell Res Ther. 2021 Jul 22;12:414. doi: 10.1186/s13287-021-02481-9 (PMC8296733; doi:10.1186/s13287-021-02481-9)
Supplement: Supplementary file 1 — Additional file 1: Fig. S1. Expression of positive and negative cell-surface markers in mesenchymal stromal cells (MSCs), and their differentiation potential. (A) Representative flow cytometric profiles of colony-forming synovium-derived MSCs stained for CD44, CD73, CD90, and CD105 (positive cell-surface markers), and CD45 and CD31(negative cell-surface markers) (purple: isotype control; red: sample). (B) Chondrogenesis. Histological sections stained with toluidine bule are shown. (C) Adipogenesis. Culture dishes stained with oil red-O are shown. (D) Calcification. Culture dishes stained with alizarin red are shown. Fig. S2. Effect of MSC-EVs on meniscus regeneration. Histological and immunohistochemical (IHC) staining of the best and the worst regenerated menisci with or without MSCs EV treatment. Samples were stained with HE, safranin-O/fast green, type II collagen (Col II), and type I collagen (Col I) (n = 6; 3 weeks). Fig. S3. Early effect of MSC-EVs in a mouse meniscal defect model. IHC evaluation of proliferative cell nuclear antigen (PCNA) staining after 1 week in the best and the worst regenerated meniscus with and without MSC-EV treatment. Fig. S4. Volcano plot presenting the transcriptome/RNA sequencing data for synovial MSCs cultured with or without MSC-EVs for 24 h (n = 4). Gene set enrichment analysis. Fig. S5. KEGG pathway analysis of synovial MSCs treated with MSC-EVs. Fig. S6. Protein-protein interactions on upregulated gene set in synovial MSCs treated with MSC-EVs. [file 13287_2021_2481_MOESM1_ESM.pptm]

## Slide 1
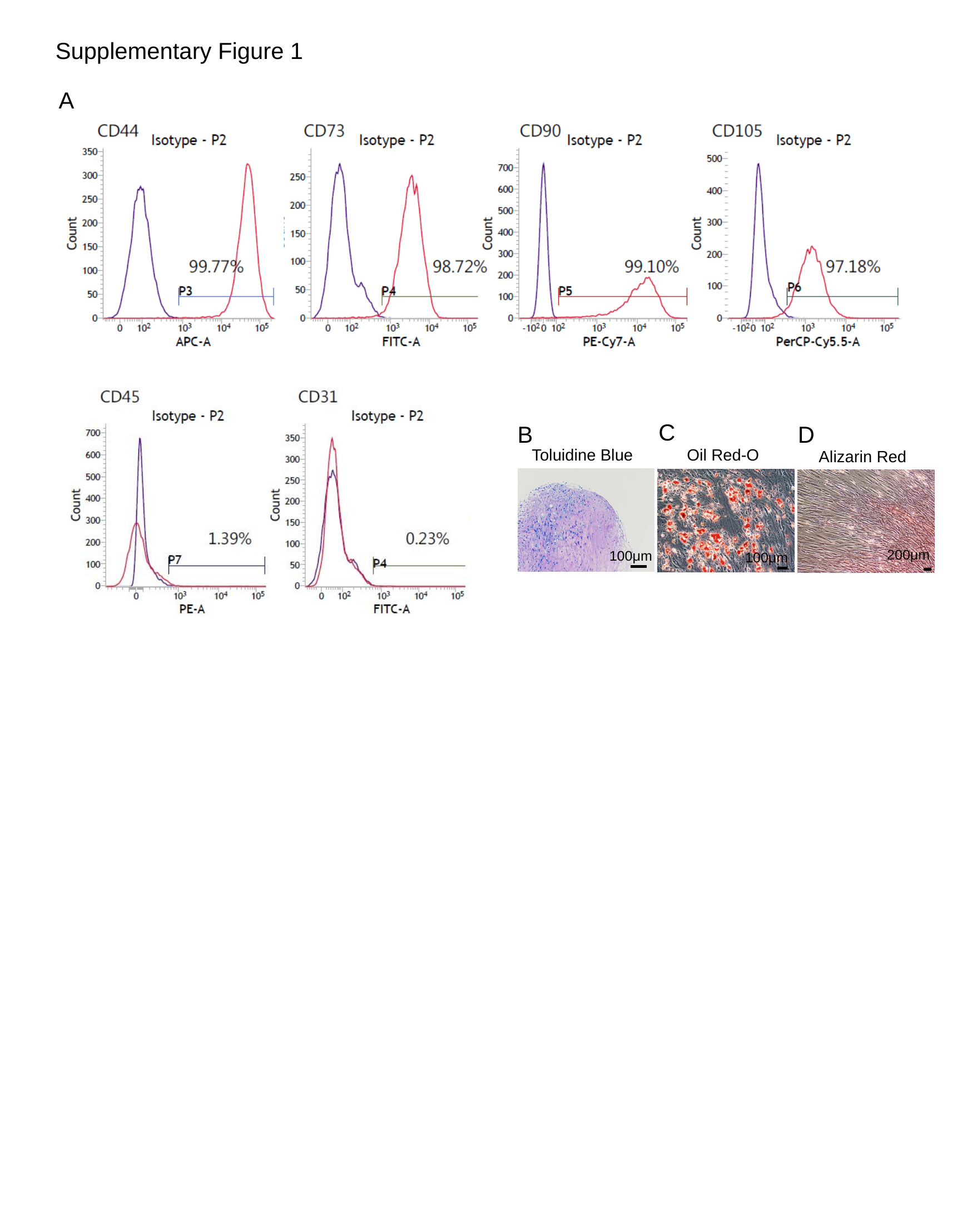

Supplementary Figure 1
A
C
B
D
Toluidine Blue
Oil Red-O
Alizarin Red
200μm
100μm
100μm

## Slide 2
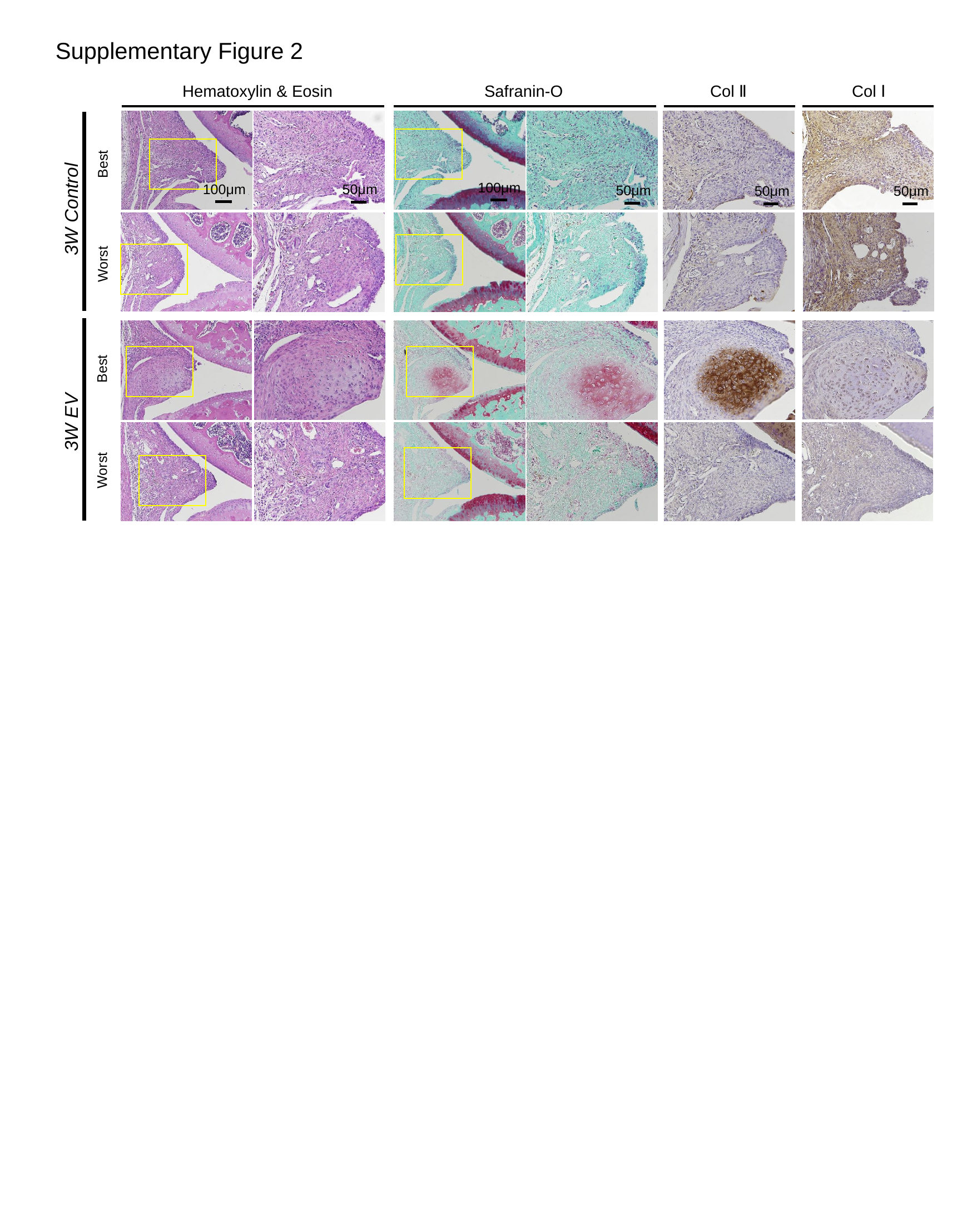

Supplementary Figure 2
Hematoxylin & Eosin
Safranin-O
Col Ⅱ
Col Ⅰ
Best
100μm
100μm
50μm
50μm
50μm
50μm
100μm
3W Control
Worst
Best
3W EV
Worst

## Slide 3
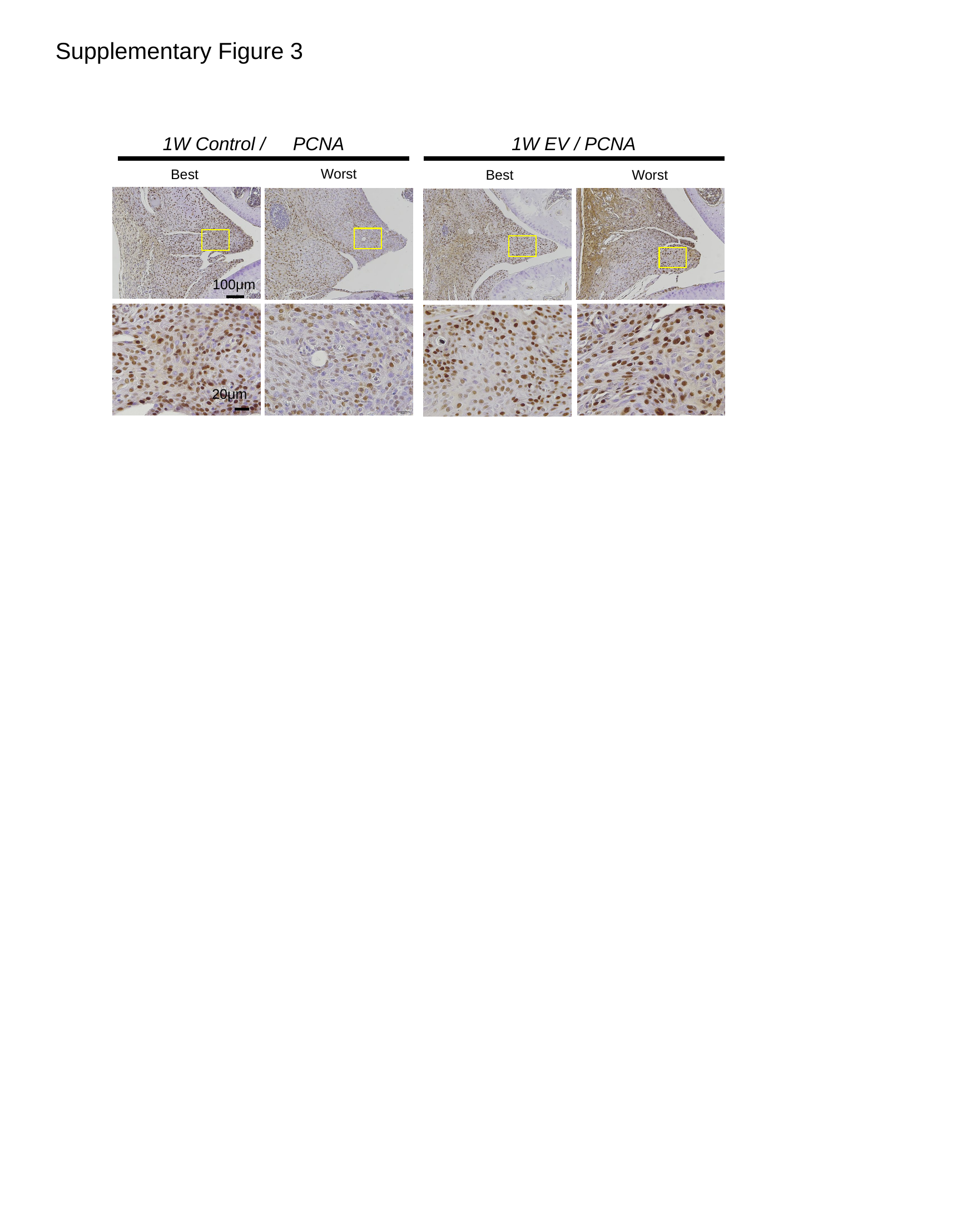

Supplementary Figure 3
1W Control /　PCNA
1W EV / PCNA
Worst
Best
Best
Worst
100μm
20μm

## Slide 4
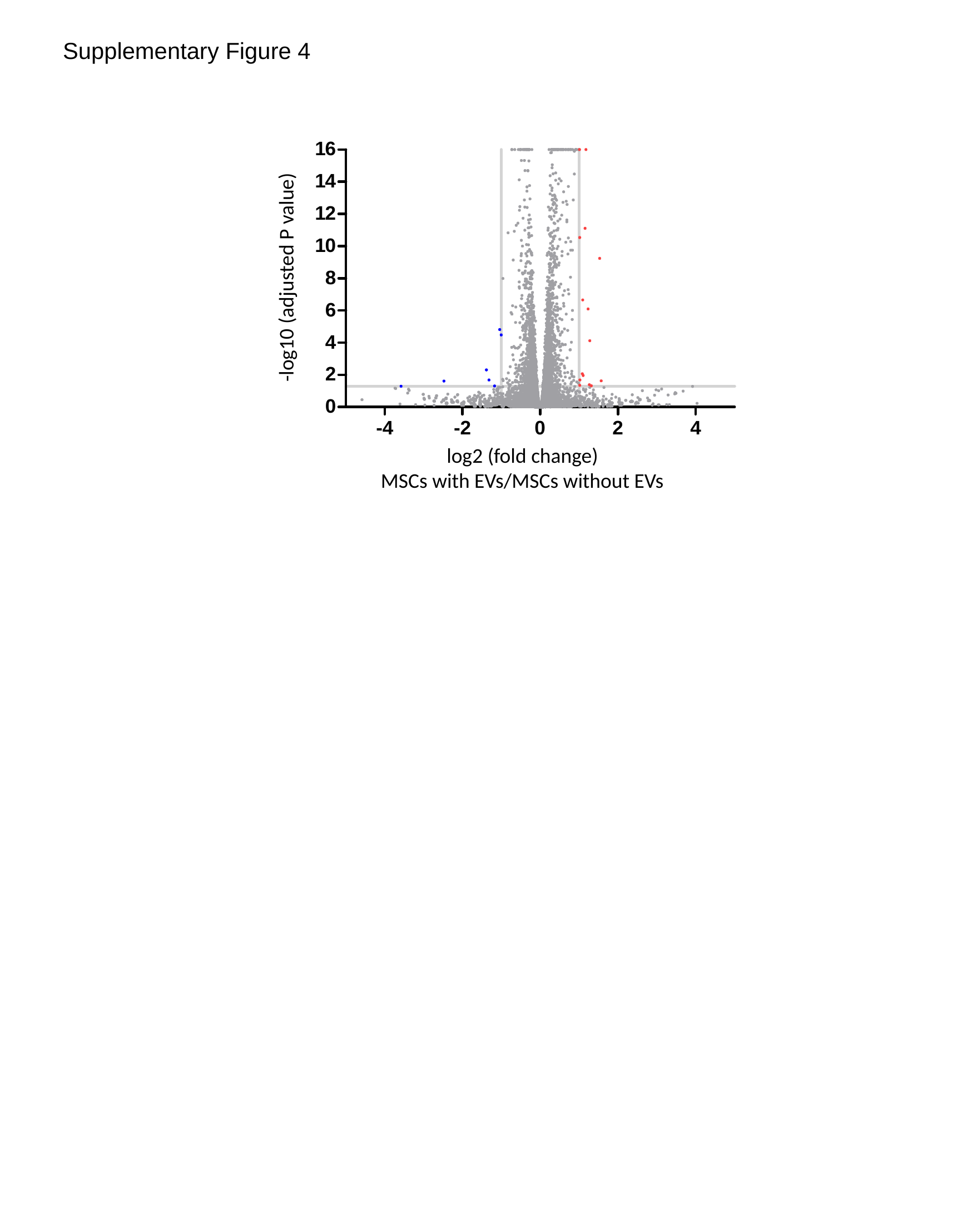

Supplementary Figure 4
-log10 (adjusted P value)
log2 (fold change)
MSCs with EVs/MSCs without EVs

## Slide 5
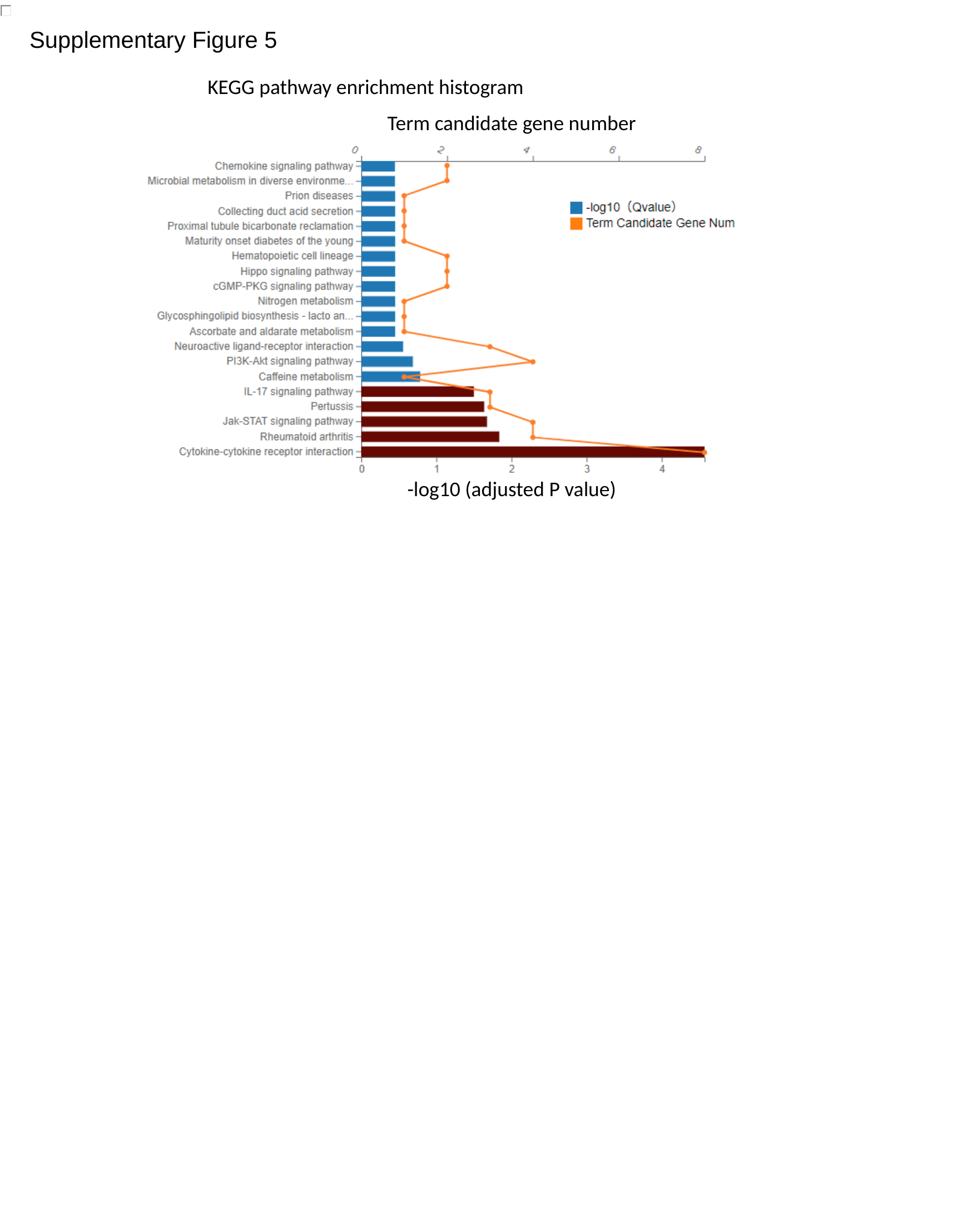

Supplementary Figure 5
KEGG pathway enrichment histogram
Term candidate gene number
-log10 (adjusted P value)

## Slide 6
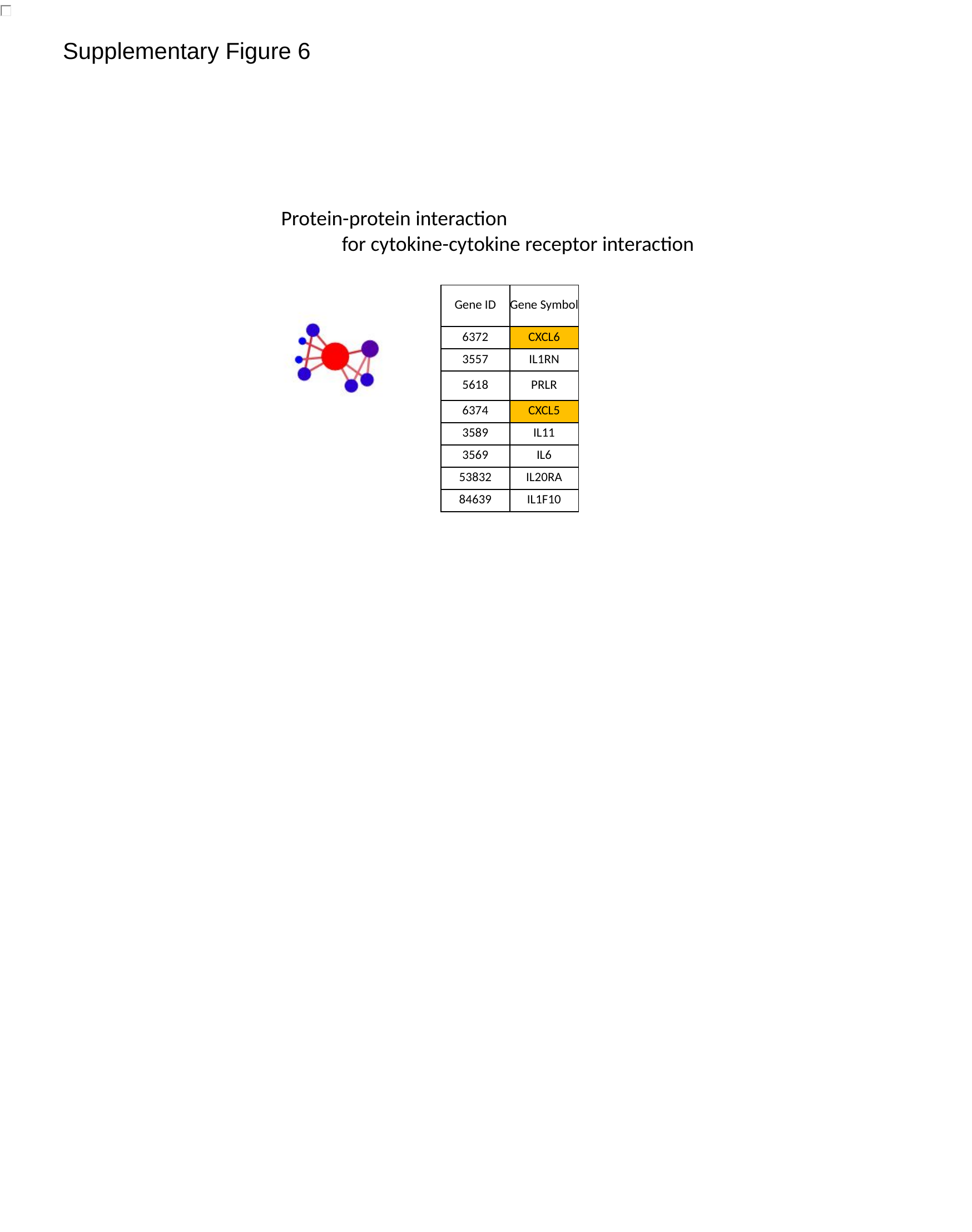

Supplementary Figure 6
Protein-protein interaction
for cytokine-cytokine receptor interaction
| Gene ID | Gene Symbol |
| --- | --- |
| 6372 | CXCL6 |
| 3557 | IL1RN |
| 5618 | PRLR |
| 6374 | CXCL5 |
| 3589 | IL11 |
| 3569 | IL6 |
| 53832 | IL20RA |
| 84639 | IL1F10 |
