# Supplementary material for: Extracellular vesicles derived from mesenchymal stromal cells mediate endogenous cell growth and migration via the CXCL5 and CXCL6/CXCR2 axes and repair menisci
Source: Stem Cell Res Ther. 2021 Jul 22;12:414. doi: 10.1186/s13287-021-02481-9 (PMC8296733; doi:10.1186/s13287-021-02481-9)
Supplement: Supplementary file 3 — Additional file 3: Table S2. Criteria and scores used for the histological assessment of regenerated menisci (Modified Pauli’s Score, A:3, B:2, C:1, D:0). [file 13287_2021_2481_MOESM3_ESM.docx]

**Supplementary table 2.** Criteria and scores used for the histological assessment of regenerated menisci (Modified Pauli’s Score, A:3, B:2, C:1, D:0)

I. Surface including lamellar layer:

I–I. Femoral side:

A Smooth

B Slight fibrillation or slightly undulating

C Moderate fibrillation or markedly undulating

D Severe fibrillation or disruption

I–II. Tibial side

A Smooth

B Slight fibrillation or slightly undulating

C Moderate fibrillation or markedly undulating

D Severe fibrillation or disruption

I–III. Inner border

A Smooth

B Slight fibrillation or slightly undulating

C Moderate fibrillation or markedly undulating

D Severe fibrillation or disruption

II. Cellularity of meniscal cell

A Normal cell distribution

B Moderately normal cell distribution

C Hypercellularity or hypocellularity

D No meniscal cells

III. Collagen fiber organization

A Collagen fibers well organized, no separations or tears

B Collagen fibers moderately organized, slight separations or tears

C Collagen fiber unorganized, moderate separations or tears

D Collagen fiber unorganized, severe separations or tears

IV. Matrix staining (safranin-O)

A Well stained like normal meniscus

B Moderately stained

C Slightly stained

D No stain
